# Supplementary material for: Multi-omics analysis indicates an association between TAPBP and prostate cancer
Source: PLoS One. 2025 Nov 21;20(11):e0336438. doi: 10.1371/journal.pone.0336438 (PMC12637951; doi:10.1371/journal.pone.0336438)
Supplement: Attachment — Submitted filename: PONE-D-24-53031_reviewer_PF.pdf [file pone.0336438.s003.pdf]

PLOS ONE

Multi-omics association study identifies potential prostate cancer- causing genes

--Manuscript Draft--

**Manuscript Number:**	PONE-D-24-53031
**Article Type:**	Research Article
**Full Title:**	Multi-omics association study identifies potential prostate cancer- causing genes
**Short Title:**	potential prostate cancer- causing genes
**Corresponding Author:**	Zhiyong Liu<br>Changhai Hospital<br>shanghai, CHINA
**Keywords:**	prostate cancer; Proteome-wide association studies (PWAS); Genome-wide association study (GWAS); Transcriptome-wide association studies (TWAS); Mendelian Randomization (MR) analysis
**Abstract:**	BACKGROUND<br><br>Prostate cancer is one of the most common malignant tumors among men worldwide, and surgery remains its mainstay of treatment. It is unclear how prostate cancer develops and what the most effective drug targets are for treating prostate cancer. Therefore, we sought to identify the genes responsible for prostate cancer.<br><br>METHODS<br><br>By integrating multidimensional and high-throughput data, proteome wide association studies (PWAS), transcriptome wide association studies (TWAS), single-cell sequencing, functional enrichment, Mendelian randomization (MR), and Bayesian co-localization analyses were used to screen for candidate genes that may contribute to prostate cancer and associate with clinical results of prostate cancer.<br><br>RESULTS<br><br>Our comprehensive analysis showed that protein abundance of eight genes was associated with prostate cancer, four of which were validated at the transcriptome level. These 8 candidate genes (MSMB, PLG, CHMP2B, ATF6B, EGF, TAPBP, GAS1 and MMP7) were validated. After combining single-cell sequencing, Mendelian randomization, and Bayesian co-localization analyses, we identified 1 gene (TAPBP) that is strongly associated with prostate cancer.
**Order of Authors:**	Xinlong Wang
	Aimin Jiang
	Lei Wang
	Zhiyong Liu
**Additional Information:**	
**Question**	**Response**
**Financial Disclosure**<br><br>Enter a financial disclosure statement that describes the sources of funding for the work included in this submission. Review the submission guidelines for detailed requirements. View published research articles from *PLOS ONE* for specific examples.	Yes

Powered by Editorial Manager® and ProduXion Manager® from Aries Systems Corporation

This statement is required for submission and **will appear in the published article** if the submission is accepted. Please make sure it is accurate.<br><br>**Funded studies**<br>Enter a statement with the following details:<br>• Initials of the authors who received each award<br>• Grant numbers awarded to each author<br>• The full name of each funder<br>• URL of each funder website<br>• Did the sponsors or funders play any role in the study design, data collection and analysis, decision to publish, or preparation of the manuscript?<br><br>Did you receive funding for this work?	
Please add funding details.<br>as follow-up to "**Financial Disclosure**<br><br>Enter a financial disclosure statement that describes the sources of funding for the work included in this submission. Review the submission guidelines for detailed requirements. View published research articles from *PLOS ONE* for specific examples.<br><br>This statement is required for submission and **will appear in the published article** if the submission is accepted. Please make sure it is accurate.<br><br>**Funded studies**<br>Enter a statement with the following details:<br>• Initials of the authors who received each award<br>• Grant numbers awarded to each author<br>• The full name of each funder<br>• URL of each funder website<br>• Did the sponsors or funders play any role in the study design, data collection and analysis, decision to publish, or preparation of the manuscript?	This work was supported by the 234 Panfeng Program at Changhai Hospital (No. 2020YXK030).

Did you receive funding for this work?"	
Please select the country of your main research funder (please select carefully as in some cases this is used in fee calculation).<br>as follow-up to "**Financial Disclosure**<br><br>Enter a financial disclosure statement that describes the sources of funding for the work included in this submission. Review the submission guidelines for detailed requirements. View published research articles from *PLOS ONE* for specific examples.<br><br>This statement is required for submission and **will appear in the published article** if the submission is accepted. Please make sure it is accurate.<br><br>**Funded studies**<br>Enter a statement with the following details:<br>• Initials of the authors who received each award<br>• Grant numbers awarded to each author<br>• The full name of each funder<br>• URL of each funder website<br>• Did the sponsors or funders play any role in the study design, data collection and analysis, decision to publish, or preparation of the manuscript?<br><br>Did you receive funding for this work?"	CHINA - CN
**Competing Interests**<br><br>Use the instructions below to enter a competing interest statement for this submission. On behalf of all authors, disclose any competing interests that could be perceived to bias this work—acknowledging all financial support and any other relevant financial or non-financial competing interests.<br><br>This statement is required for submission	The authors declare that they have no competing interests.

and **will appear in the published article** if the submission is accepted. Please make sure it is accurate and that any funding sources listed in your Funding Information later in the submission form are also declared in your Financial Disclosure statement.<br><br>View published research articles from *PLOS ONE* for specific examples.<br><br>**NO authors have competing interests**<br><br>Enter: *The authors have declared that no competing interests exist.*<br><br>**Authors with competing interests**<br><br>Enter competing interest details beginning with this statement:<br><br>*I have read the journal's policy and the authors of this manuscript have the following competing interests: [insert competing interests here]*<br><br>* typeset	
**Ethics Statement**<br><br>Enter an ethics statement for this submission. This statement is required if the study involved:<br><br>• Human participants<br>• Human specimens or tissue<br>• Vertebrate animals or cephalopods<br>• Vertebrate embryos or tissues<br>• Field research<br><br>Write "N/A" if the submission does not require an ethics statement.<br><br>General guidance is provided below. Consult the submission guidelines for detailed instructions. **Make sure that all information entered here is included in the Methods section of the manuscript.**	This study does not involve direct research on humans or animals.

**Format for specific study types**<br><br>**Human Subject Research (involving human participants and/or tissue)**<br>• Give the name of the institutional review board or ethics committee that approved the study<br>• Include the approval number and/or a statement indicating approval of this research<br>• Indicate the form of consent obtained (written/oral) or the reason that consent was not obtained (e.g. the data were analyzed anonymously)<br><br>**Animal Research (involving vertebrate animals, embryos or tissues)**<br>• Provide the name of the Institutional Animal Care and Use Committee (IACUC) or other relevant ethics board that reviewed the study protocol, and indicate whether they approved this research or granted a formal waiver of ethical approval<br>• Include an approval number if one was obtained<br>• If the study involved *non-human primates*, add *additional details* about animal welfare and steps taken to ameliorate suffering<br>• If anesthesia, euthanasia, or any kind of animal sacrifice is part of the study, include briefly which substances and/or methods were applied<br><br>**Field Research**<br><br>Include the following details if this study involves the collection of plant, animal, or other materials from a natural setting:<br>• Field permit number<br>• Name of the institution or relevant body that granted permission	
**Data Availability**<br><br>Authors are required to make all data underlying the findings described fully available, without restriction, and from the time of publication. PLOS allows rare exceptions to address legal and ethical concerns. See the PLOS Data Policy and FAQ for detailed information.	Yes - all data are fully available without restriction

A Data Availability Statement describing where the data can be found is required at submission. Your answers to this question constitute the Data Availability Statement and **will be published in the article**, if accepted.<br><br>**Important:** Stating 'data available on request from the author' is not sufficient. If your data are only available upon request, select 'No' for the first question and explain your exceptional situation in the text box.<br><br>Do the authors confirm that all data underlying the findings described in their manuscript are fully available without restriction?	
**Describe where the data may be found in full sentences. If you are copying our sample text, replace any instances of XXX with the appropriate details.**<br><br>• If the data are **held or will be held in a public repository**, include URLs, accession numbers or DOIs. If this information will only be available after acceptance, indicate this by ticking the box below. For example: *All XXX files are available from the XXX database (accession number(s) XXX, XXX.)*.<br>• If the data are all contained **within the manuscript and/or Supporting Information files**, enter the following: *All relevant data are within the manuscript and its Supporting Information files.*<br>• If neither of these applies but you are able to provide **details of access elsewhere**, with or without limitations, please do so. For example:<br><br>*Data cannot be shared publicly because of [XXX]. Data are available from the XXX Institutional Data Access / Ethics Committee (contact via XXX) for researchers who meet the criteria for access to confidential data.*<br><br>*The data underlying the results presented in the study are available from (include the name of the third party*	All relevant data are within the manuscript and its Supporting Information files.

and contact information or URL).<br>• This text is appropriate if the data are owned by a third party and authors do not have permission to share the data.<br><br>* typeset	
Additional data availability information:	Tick here if your circumstances are not covered by the questions above and you need the journal's help to make your data available.

Powered by Editorial Manager® and ProduXion Manager® from Aries Systems Corporation

Click here to access/download;Manuscript;manuscript.docx

Multi-omics association study identifies potential prostate cancer- causing genes

Xinlong Wang [1, †], Aimin Jiang[1,†] , Lei Wang[1,*], Zhiyong Liu[1,*]

[1 1] Department of Urology, Changhai Hospital, Naval Medical University, Shanghai, China

[†]These authors contributed equally

Abstract

BACKGROUND: Prostate cancer is one of the most common malignant tumors among men worldwide, and surgery remains its mainstay of treatment. It is unclear how prostate cancer develops and what the most effective drug targets are for treating prostate cancer. Therefore, we sought to identify the genes responsible for prostate cancer.

METHODS: By integrating multidimensional and high-throughput data, proteome wide association studies (PWAS), transcriptome wide association studies (TWAS), single-cell sequencing, functional enrichment, Mendelian randomization (MR), and Bayesian co-localization analyses were used to screen for candidate genes that may contribute to prostate cancer and associate with clinical results of prostate cancer.

RESULTS: Our comprehensive analysis showed that protein abundance of eight genes was associated with prostate cancer, four of which were validated at the transcriptome level. These 8 candidate genes (MSMB, PLG, CHMP2B, ATF6B, EGF, TAPBP, GAS1 and MMP7) were validated. After combining single-cell sequencing, Mendelian randomization, and Bayesian co-localization analyses, we identified 1 gene (TAPBP) that is strongly associated with prostate cancer.

Key words: prostate cancer, Proteome-wide association studies (PWAS), Genome-wide association study (GWAS), Transcriptome-wide association studies (TWAS), Mendelian Randomization (MR) analysis.

1 Introduction

Prostate cancer (PCa) is one of the most common malignancies among men worldwide. According to Global Cancer Statistics 2020, PCa is the leading cause of cancer-related death in men [1]. Numerous PCa-associated risk loci have been screened using the genome-wide association study (GWAS) approach [2]. GWAS aims to tightly link genetic loci to diseases and other genetic traits [3, 4]. Over the past decade, the approach has involved many variant-phenotype associations [5] and has driven important scientific discoveries [6].

Despite the tremendous impact of GWAS, inherent difficulties continue to limit its success [7, 8]. An improvement of these methods would be a protein-centered approach that considers the impact of genetic variation on gene function. Proteins are the most effective biomarkers and therapeutic targets [9, 10] because they represent the major functional components of cellular and biological processes as well as the end products of gene expression [11]. The study of risk proteins in PCa is crucial. Overall, studies have shown that alterations in proteomics are associated with cell cycle control, DNA repair, proteasome degradation and metabolic activity. In addition to differences in study methodology, possibly due to the heterogeneity of PCa itself, the results are rather inconsistent [12].

Based on the available literature, we used proteome wide association studies (PWAS). PWAS is based on the premise that causal variation in coding regions affects phenotype by altering the biochemical function of a gene's protein product. To capture these effects, PWAS quantifies the extent to which proteins are impaired under individual genotypes.

Like other gene-based approaches, PWAS alleviates the burden of multiple test corrections. In addition, it provides specific functional explanations for the protein-coding genes it discovers. Therefore, we sought to identify new drug targets for the treatment of PCa by combining high-throughput proteomics in PCa with genetic data to determine the levels of genomic structure-associated proteins. To identify potential protein biomarkers, we took a six-step approach to systematically link protein biomarkers to PCa. First, we used findings from the Atherosclerosis Risk in Communities (ARIC) dataset and prostate cancer GWAs to perform PWAS and its FDR-corrected analysis. Next, we analyzed the expression of PWAS-identified risk genes by the Prostate Cancer Single Cell Database and explore the potential function of candidate genes. The we explored

important genes driving PWAS signaling at the transcriptional level by utilizing gene expression data. Fourth we used independent Mendelian randomization (MR) analysis to validate PWAS significant genes and used COLOC to integrate the GWAs data with the PCa database using Bayesian co-localization analysis to explore whether the two correlated signals were consistent with a common dependent variable(s). Fifth, we explored the mutation and drug sensitivity of TAPBP. Finally, we explored the association of these potential prostate cancer-causing proteins with some PCa clinical features.

2 Method

2.1 Source of data

2.1.1 Source of GWAS data

A large number of meta-analyses have been conducted on PCa GWAS, including the UK biobank (UKB) data are fully available at http://fastgwa.info/ukbimpbin and the GWAS Catalog. According to UKB and GWAS Catalog summary statistics, 7769 cases and 201039 controls were considered eligible. All participants for PCa included were of European descent.

2.1.2 Human blood proteomic and transcriptomic data

Serum proteomic data were collected Atherosclerosis Risk in Communities (ARIC) study; N~9,000)[13] in a large population-based study. The ARIC study enrolled 15,792 participants from four American communities. The current study ultimately included 9,084 participants with plasma protein data. A total of 4,657 human serum proteins were considered eligible.

Local gene expression regulation is often shared across tissues [14, 15]and combining eQTL data across multiple tissues can improve the power of TWAS. Thus, Genotype-Tissue Expression (GTEx) version 8 data (n=948) was used for the analysis of whole blood eQTLs [16]. A paired RNA-seq (Illumina TruSeq; Illumina Inc) was used to obtain gene expression data, and whole genome sequencing was used to obtain genotype data. The GTEx website provides information on donor registration, consent procedures, methods for obtaining biological samples, sample attachments, and procedures for histopathological examinations [17].

2.1.3 Single cell data

Single cell datasets of PCa were downloaded from GEO with the access number of GSE141445. The pre-processing standards for Seurat object were as follow: number of RNA count >= 1000, number of features >= 200, percent.mt <= 20, percent.rb<= 20. Double cells were removed by R package DoubletFinder with default parameters.

2.2 Statistical analysis

2.2.1 Proteome-wide association studies (PWAS)

The overall design of this study is shown in **Figure 1**. The effect of SNPs on protein abundance was calculated for proteins with significant heritability (P < 0.01) using FUSION [18]. The common prediction models used in the analysis are top1, blup, lasso, enet and bslmmv [18]. the ARIC database was selected for this analysis, so only enet and top1 were selected for the analysis. [13]. To combine the genetic effects of PCa with protein weights for PWAS of PCa, a linear sum of Z-scores + weights of independent SNPs was calculated. To reduce the likelihood of false positives, a Bonferroni-corrected P-value threshold was employed. Benjamini-Hochberg (BH) was also used to impute the P value adjusted for false discovery rate (FDR).

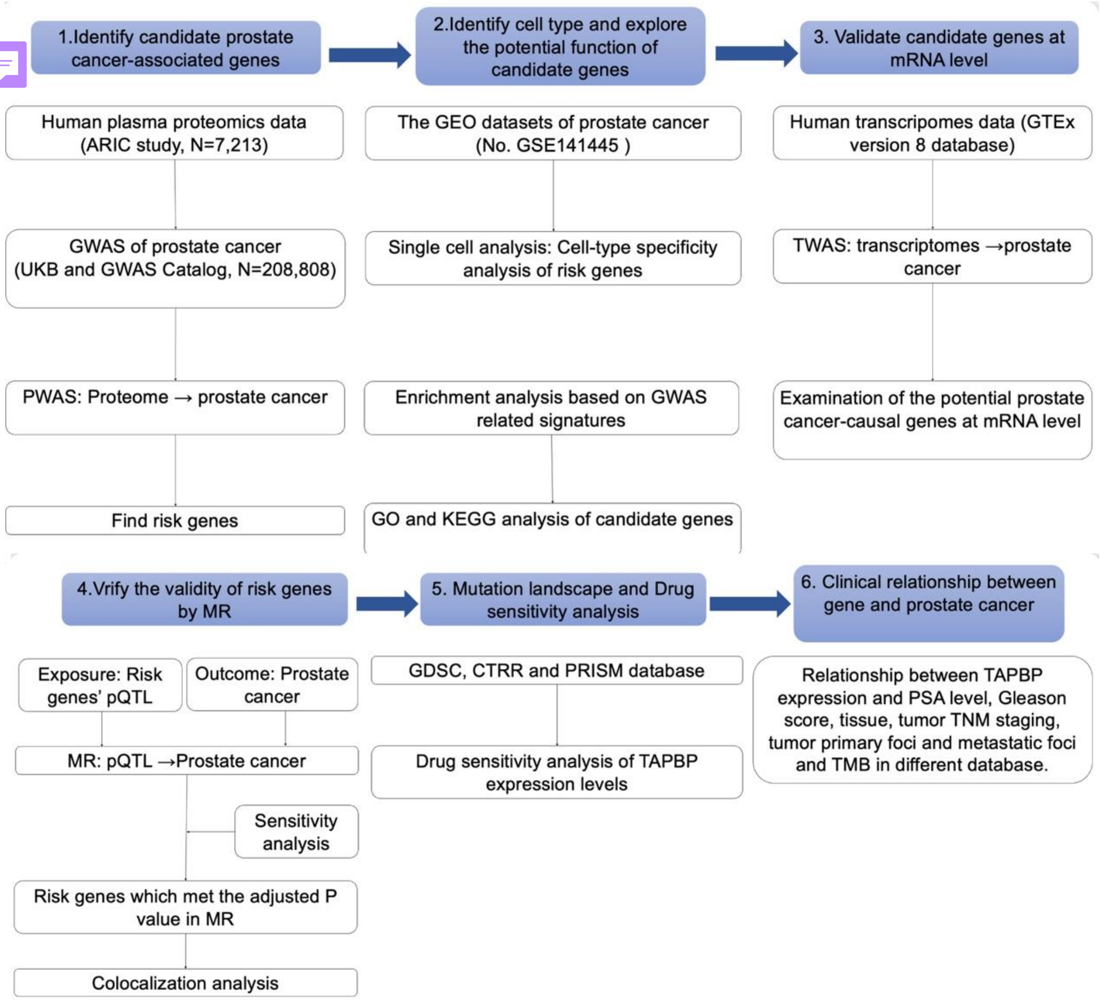

Fig 1. Overview of this study

2.2.2 Single cell analysis

R package harmony was applied to remove batch effect of samples from different patients. Cell type identification was determined by known marker genes and aid of R package SingleR. Enrichment score of GWAS related signatures for each cell were analyzed by function AddModuleScore from R package Seurat. More information about scRNA sequencing library preparation and sequence strategy can be found in work from study. In addition, more detailed parameters and pipelines for single cell analysis could refer to our previous works [19, 20, 21]

2.2.3 Transcriptome-wide association studies (TWAS)

The genetic influence of PCa was integrated with mRNA expression weights by using FUSION [18]. The basic procedure is as follows: Firstly, TWAS expression weights (i.e. SNP-gene correlations)

are calculated from a reference expression panel [22]. To determine the out-of-sample R2 of each gene prediction model, FUSION performed five-fold cross-validation [18].

2.2.4 Mendelian Randomization (MR) analysis

The PWAS significant genes (from the FUSION method) were examined for cis-regulatory protein abundance using MR. The genome-wide significant ($P < 5 \times 10^{-6}$) SNPs were considered as inclusion and followed by linkage disequilibrium (LD) to screen for independent SNPs ($R2 < 0.1$). A harmonization of exposure (QTL) and outcome (PCa GWAS) data was then performed. Wald ratios can be used to estimate causality when only one independent QTL is available. In cases where multiple SNPs are available, the ratios of SNP exposures to SNP outcomes were estimated using the inverse variance weighting (IVW) method for random-effects meta-analysis. In addition, when the number of SNPs exceeded three, horizontal pleiotropy was tested using the MR-egger method. Bonferroni correction thresholds for the number of genes analyzed were set at $P < 0.05$/multiple comparisons [23, 24]. Mendelian randomization analysis was conducted using "TwoSampleMR" version 0.5.5 in R version 4.0.

2.2.5 Bayesian colocalization analysis

The Coloc Bayesian test was used to determine the probability that the PCa risk locus and the pQTL share the same causal signal [25, 26]. In prostate disease, p1 represents the probability that a specific variant correlates with a significant pQTL, p2 represents the likelihood that a particular variant will be associated with one in the disease. An individual variant's p12 is the probability of it being both an outcome and a pQTL in prostate disease. Five mutually exclusive hypotheses were tested: H0, no relation with either GWAS or pQTL; H1, relation with GWAS and no relation with pQTL; H2, relation with pQTL and no relation with GWAS; H3, relation with GWAS and pQTL, two independent SNPs; and H4, association with GWAS and pQTL, one common SNP. We focus on the last hypothesis, H4, and the posterior probability (PP) is employed to support H4 (denoted as PPH4). We define strong proof for co-localization when $PPH4 \geq 0.75$.

2.2.6 Enrichment analysis based on candidate genes

Firstly, bulk sequencing datasets including ICGC-PRAD, TCGA-PRAD, GSE21034, GSE54460,

GSE107299, GSE70768, GSE70769, GSE21034, DFZ2018 were downloaded from with the use of R packages TCGAbiolinks and GEOquary [27]. All transcriptome or microarray matrix were scaled and normalized before formal analysis. In addition, patients were excluded if missing clinical outcome information. Next, an enrichment score for each patient were calculated by R package GSVA with the function of ssGSEA [28]. Next, the biological annotation and hallmarks enrichment score correlated with score of discovery PWAS genes were analyzed by R packages clusterprofile and ggpubr, which was verified by algorithm of rank-based analysis (ORA).

2.2.7 Analysis of drug sensitivity analysis and mutation profile

Three drug sensitivity datasets, GDSC, CTRR and PRISM, were enrolled to detect the influence of GWAS score on therapy response or resistance by R package pRRophetic [29, 30].

All visualization were performed with the application of software including R and GraphPad. This study examined the clinical disparities of GSWA score using student T and Wilcoxon tests. The R package ggpubr was employed to assess the different GWAS score across different T, Stage, and Grade classifications through the Kruskal-Wallis test.

3 Result

3.1 Discovery of PWAS in PCa

The FUSION pipeline was used to integrate the PCa GWAS findings with human blood proteomes for the PWAS of PCa. Based on the 9084-master analyzed sample size derived from the ARIC database, the PWAS identified 16 genes (MSMB, PLG, CHMP2B, ATF6B, EGF, TAPBP, GAS1, MMP7, SERPINA3, AIF1, PRDX3, DKK3, PSAPL1, MINPP1, ANGPTL4, and CTSS), whose protein levels were associated with PCa at $P<0.05$ (**Figure 2**). After Bonferroni correction threshold of $P < 0.05$/number, where Microseminoprotein-beta (MSMB), Plasminogen (Plg), CHMP2B, Activating transcription factor 6 (ATF6), Epidermal Growth Factor (EGF), Tapasin Binding Protein (TAPBP), Growth Arrest Specific Gene 1 (GAS1) and matrix metalloproteinase 7 (MMP7) were more significantly associated with PCa.

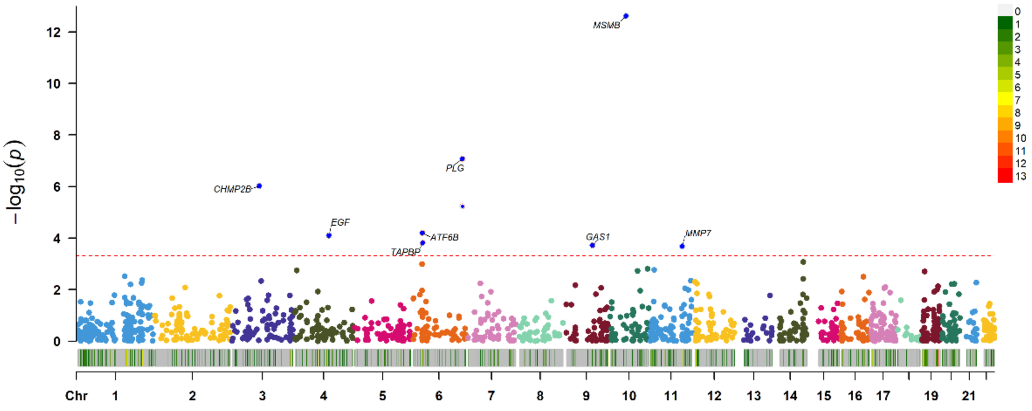

Fig 2. Manhattan plot for the discovery PCa PWAS integrating the PCa GWAS (N = 7769) with the discovery ARIC proteomes (N = 15792). Each point represents a single association test between a gene and lacunar stroke ordered by genomic position on the x axis and the association strength on the y axis as the $-\log_{10}(P)$ of a z-score test. The discovery PWAS identified 8 genes whose cis-regulated protein abundance was associated with PCa at an FDR of P < 0.05. The red horizontal line reflects the significant threshold of the FDR P < 0.05 and is set at the highest unadjusted P value that is below that threshold ($P = 2.2 \times 10^{-4}$)

3.2 Expression of these genes in different cell types of PCa

We investigated whether risk genes identified by PWAS are enriched in specific prostate cell types. Using human single-cell RNA-seq data from a cell-type database, we identified enriched causal genes expressed specifically in eight cell types (**Figure 3**). TAPBP, ATF6B, CHMP2B, MSMB were expressed in a higher proportion of malignant cells, while TAPBP was expressed in a higher proportion and level in various immune cells.

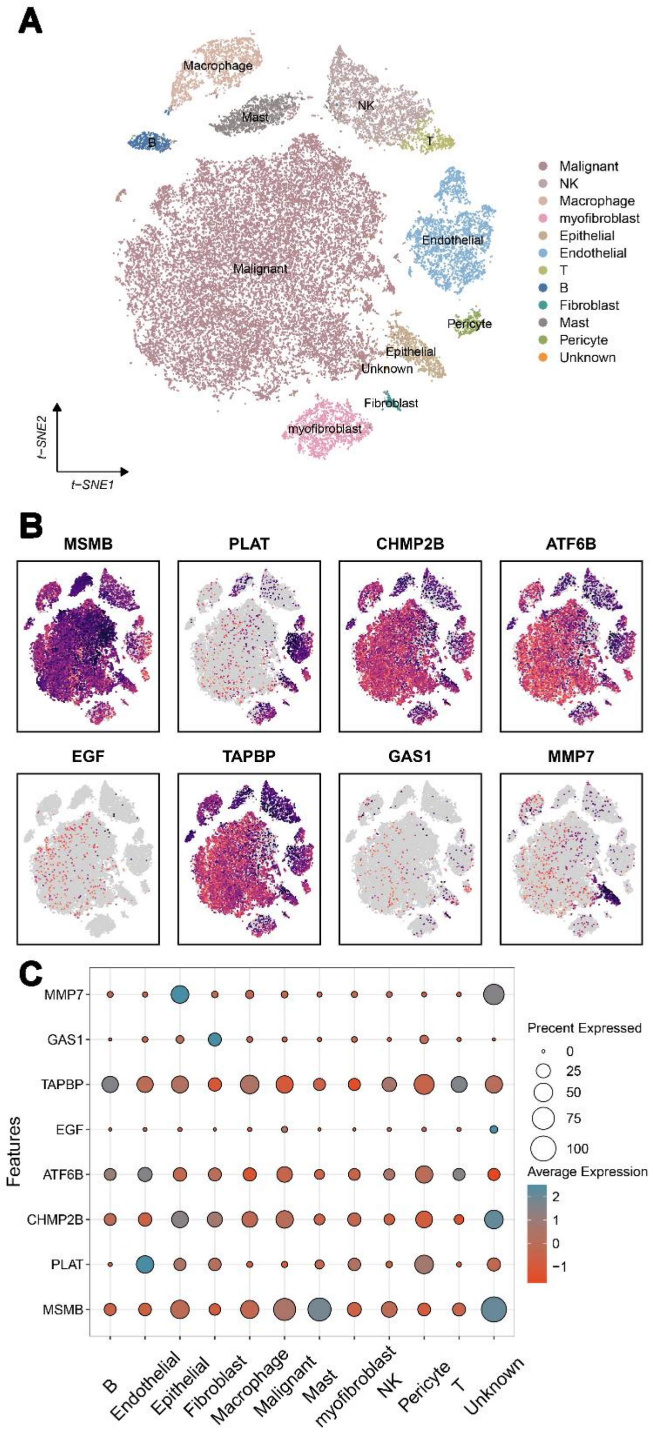

Fig 3. Expression of 8 genes in PCa tissues. **A**. Cell types in PCa tissues. **B, C**. Expression profiles of 8 genes in PCa tissues

3.3 Enrichment Analysis of the genes in PCa

In addition, we utilized the overrepresentation analysis (ORA) algorithm to decipher the role of the PWAS discovery genes across different PCa cohorts. The GO results illustrated that PWAS

discovery genes participated in cell adhesion, response to stimulus and nuclear division in BP; extracellular exosome, extracellular organelle and centromeric region in CC; and signaling receptor binding, protein−containing complex binding, and cell adhesion molecule binding in MF (**Figure 4A**). KEGG analysis revealed that PWAS discovery genes might be involved in cell cycle, and p53 signaling pathway (**Figure 4B**). GSEA and hallmark analysis also confirmed that PWAS discovery genes mainly participated in immune response, antigen processing and presentation, T cell receptor signaling pathway and Tnfa signaling via nfkb (**Figure 4C, D**). Through the above finding, we found that PWAS discovery genes were involved in the immune response and cell cycle. Integrating the aforementioned results, we find that TAPBP is positive in all outcomes. Therefore, we will conduct further analysis on TAPBP.

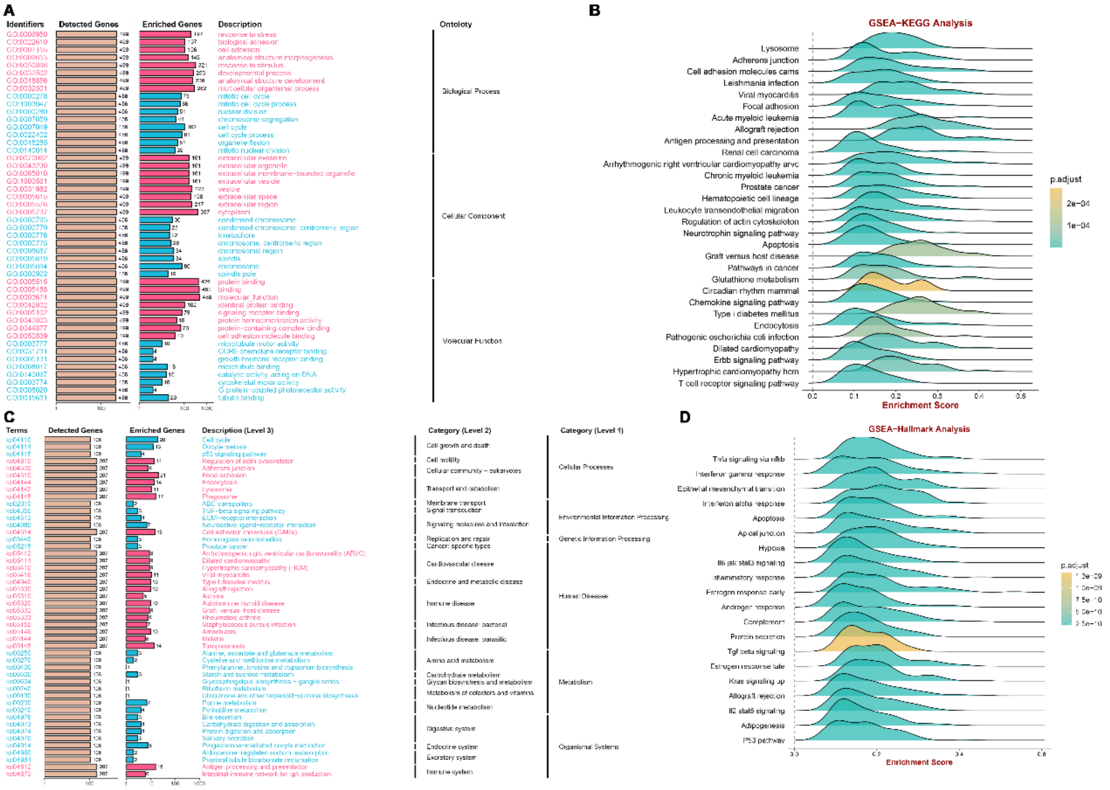

Fig 4. Candidate genes impacted signature in PCa based on correlation analysis. **A**, **B**. GO and KEGG analyses based on ORA. **C**, **D**. GSEA-KEGG, and GSEA-Hallmarks analysis of candidate genes in PCa.

3.4 TWAS identified four genes associated with PCa

We combined PCa GWAS data with the human prostate transcriptome to perform transcriptome-

wide association analysis (TWAS) of PCa using FUSION. Expression of 4 out of 8 genes (CHMP2B, ATF6B, TAPBP, GAS1) in blood was associated with PCa (**Table 1**). This suggests that the combined evidence from PWAS and TWAS suggests a role in the pathogenesis of PCa.

Table 1. TWAS identified four genes associated with Pca

gene	P value
CHMP2B	3.66E-04
ATF6B	1.68E-04
TAPBP	3.72E-03
GAS1	3.85E-01

3.5 8 genes associated with PCa were validated by MR using prostate pQTL.

Most of the analyzed proteins could only be detected using a single SNP; therefore, MR estimation was mainly based on the Wald ratio method. We further identified the above eight proteins, and these biomarkers revealed significant evidence of association in PCa GWAS (**Table 2**). We further analyzed their odds ratios and showed that among them, ATF6B, CHMP2B, GAS1, and MMP7 were the risk factors for the occurrence of PCa, while EGF, MSMB, PLG, TAPBP were protective factors (**Figure 5**).

Table 2. Candidate genes identified by Mendelian randomization.

Gene	nsnps	IVW				MR Egger	Weighted
		OR	ORLower	ORUpper	Pval	pval	median pval
ATF6B	11	1.2966	1.0964	1.5334	0.002397	0.630797082	0.000124905
CHMP2B	21	1.1427	1.0800	1.2091	3.63E-06	0.160124428	2.18935E-05
EGF	12	0.8494	0.7963	0.9061	7.21E-07	0.020274016	2.29141E-05
GAS1	4	1.2076	1.0588	1.3773	0.004923	0.774952735	0.096094973
MMP7	20	1.0962	1.0434	1.1517	0.000265	0.49333611	0.010928317
MSMB	7	0.7767	0.6945	0.8688	9.74E-06	0.327374195	0.000347019
PLG	23	0.8492	0.8066	0.8940	4.61E-10	0.017827813	2.70502E-07
TAPBP	24	0.8942	0.8620	0.9276	2.35E-09	0.004858965	1.9132E-07

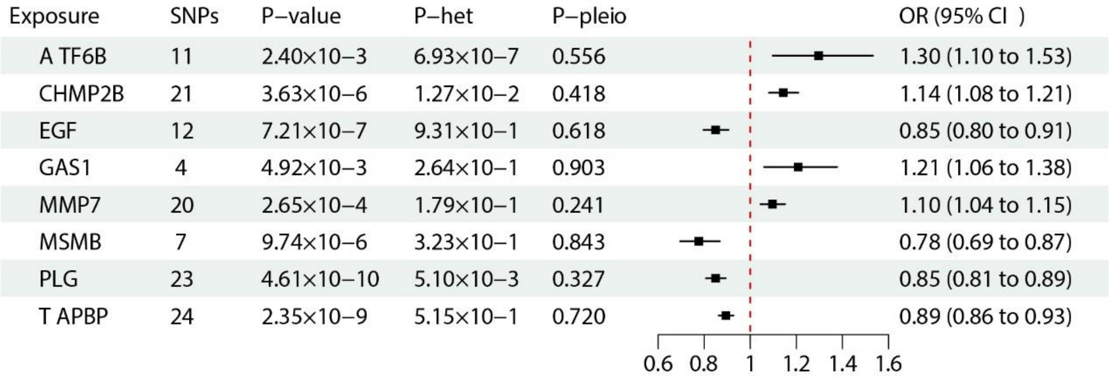

Fig 5. MR forest plot of discovered PWAS PCa-associated proteins. The results are derived from the IVW analyses

3.6 Co-localization between PCa risk genes and pQTL in the prostate gland

Prostate cancer PWAS associations may arise from coincidental overlap between pQTL and loci that are in linkage disequilibrium with PCa GWAS loci, or from the concurrent occurrence of a variant associated with protein expression, which is a protein quantitative trait locus (pQTL), and PCa. Statistical co-localization analyses for each gene report the probability of GWAS and pQTL

sharing a causal variant, referred to as Hypothesis 4 (H4) and PP4/(PP3+PP4) ≥ 0.75. Based on H4 ≥ 75% and PP4/(PP3+PP4) ≥ 0.75, the analysis revealed three of the eight genes that provided evidence of genetic co-localization (MSMB, TAPBP, and EGF) (**Table 3**). This suggests that these three proteins play an important role in the pathophysiology of PCa. TAPBP showed positive results in all of the above analyses, so its relationship with PCa needs to be further explored.

Table 3: Candidate genes identified by Bayesian colocalization.

Gene	nsnps	PP.H3.abf	PP.H4.abf
MSMB	596	0.000521504	0.99947791
TAPBP	5290	0.022416109	0.96049176
EGF	2841	0.079297104	0.88166609
CHMP2B	3177	0.99938787	0.00060374
GAS1	3039	0.063961688	0.46131011
MMP7	2986	0.39553352	0.02078762
PLG	811	0.994315549	0.00565747

3.7 Mutation landscape between subtypes

The detailed genomic landscape difference between subgroups is depicted in **Figure 6A**, which indicates that the low TAPBP led to a high mutation frequency of speckle type BTB/POZ protein, SPOP, gain of 8q24.21 and 11q13.2 chromosome, and loss of 19q13.2 and 19q13.2 chromosome.

3.8 Drug sensitivity analysis

We harnessed three comprehensive drug sensitivity databases, GDSC, CTRP , and PRISM, to detect the relationship between TAPBP expression and therapy sensitivity or resistance. As **Figure 6B** illustrated, high FDX1 expression displayed a consensus result of therapy resistance to Amuvatinib, Trichostatin, Pilaralisib, EHT−1864, and Panobinostat in the GDSC database; MK−2206, neuronal

differentiation inducer III, lomeguatrib, BRD−A02303741:carboplatin (1:1 mol/mol), and necrostatin−7 in the CTRP database; broxaterol, albuterol, GSK1904529A, pimobendan, thiamphenicol, eprinomectin, etc., in the PRISM database.

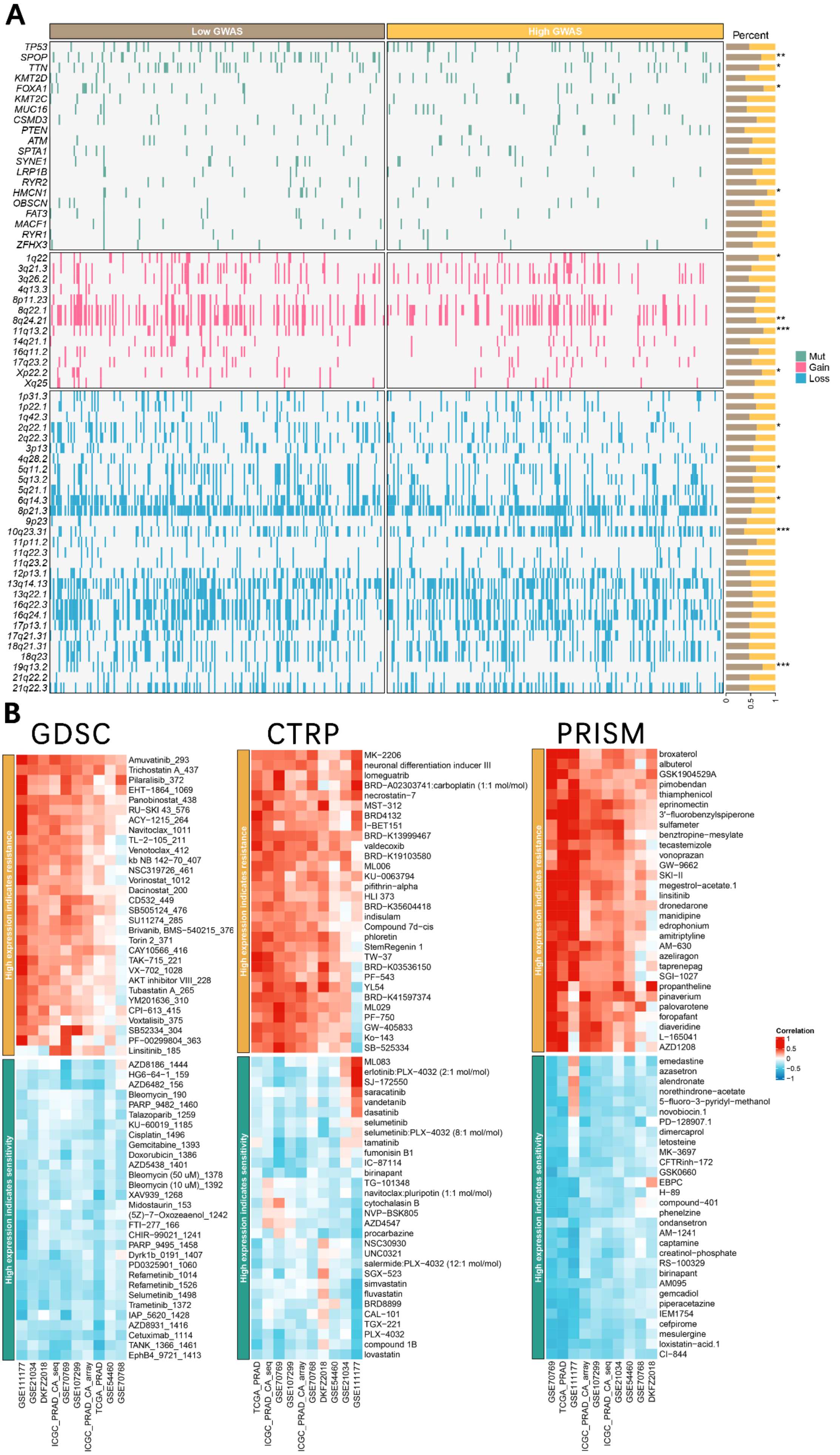
A
Low GWAS
High GWAS
Percent
TP53
SPOP
TTN
KMT2D
FOXA1
KMT2C
MUC16
CSMD3
PTEN
ATM
SPTA1
SYNE1
LRP1B
RYR2
HMCN1
OBSCN
FAT3
MACF1
RYR1
ZFHX3
1q22
3q21.3
3q26.2
4q13.3
8p11.23
8q22.1
8q24.21
11q13.2
14q21.1
16q11.2
17q23.2
Xp22.2
Xq25
1p31.3
1p22.1
1q42.3
2q22.1
2q22.3
3p13
4q28.2
5q11.2
5q13.2
5q21.1
6q14.3
8p21.3
9p23
10q23.31
11p11.2
11q22.3
11q23.2
12p13.1
13q14.13
13q22.1
16q22.3
16q24.1
17p13.1
17q21.31
18q21.31
18q23
19q13.2
21q22.2
21q22.3
Mut
Gain
Loss
0
0.5
1
B
GDSC
CTRP
PRISM
High expression indicates resistance
High expression indicates sensitivity
Amuvatinib_293
Trichostatin A_437
Pilaralisib_372
EHT−1864_1069
Panobinostat_438
RU−SKI 43_576
ACY−1215_264
Navitoclax_1011
TL−2−105_211
Venotoclax_412
kb NB 142−70_407
NSC319726_461
Vorinostat_1012
Dacinostat_200
CD532_449
SB505124_476
SU11274_285
Brivanib, BMS−540215_376
Torin 2_371
CAY10566_416
TAK−715_221
VX−702_1028
AKT inhibitor VIII_228
Tubastatin A_265
YM201636_310
CPI−613_415
Voxtalisib_375
SB52334_304
PF−00299804_363
Linsitinib_185
AZD8186_1444
HG6−64−1_159
AZD6482_156
Bleomycin_190
PARP_9482_1460
Talazoparib_1259
KU−60019_1185
Cisplatin_1496
Gemcitabine_1393
Doxorubicin_1386
AZD5438_1401
Bleomycin (50 uM)_1378
Bleomycin (10 uM)_1392
XAV939_1268
Midostaurin_153
(5Z)−7−Oxozeaenol_1242
FTI−277_166
CHIR−99021_1241
PARP_9495_1458
Dyrk1b_0191_1407
PD0325901_1060
Refametinib_1014
Refametinib_1526
Selumetinib_1498
Trametinib_1372
IAP_5620_1428
AZD8931_1416
Cetuximab_1114
TANK_1366_1461
EphB4_9721_1413
GSE111177
GSE21034
DKFZ2018
ICGC_PRAD_CA_seq
GSE70769
GSE107299
ICGC_PRAD_CA_array
TCGA_PRAD
GSE54460
GSE70768
MK−2206
neuronal differentiation inducer III
lomeguatrib
BRD−A02303741:carboplatin (1:1 mol/mol)
necrostatin−7
MST−312
BRD4132
I−BET151
BRD−K13999467
valdecoxib
BRD−K19103580
ML006
KU−0063794
pifithrin−alpha
HLI 373
BRD−K35604418
indisulam
Compound 7d−cis
phloretin
StemRegenin 1
TW−37
BRD−K03536150
PF−543
YL54
BRD−K41597374
ML029
PF−750
GW−405833
Ko−143
SB−525334
ML083
erlotinib:PLX−4032 (2:1 mol/mol)
SJ−172550
saracatinib
vandetanib
dasatinib
selumetinib
selumetinib:PLX−4032 (8:1 mol/mol)
tamatinib
fumonisin B1
IC−87114
birinapant
TG−101348
navitoclax:pluripotin (1:1 mol/mol)
cytochalasin B
NVP−BSK805
AZD4547
procarbazine
NSC30930
UNC0321
salermide:PLX−4032 (12:1 mol/mol)
SGX−523
simvastatin
fluvastatin
BRD8899
CAL−101
TGX−221
PLX−4032
compound 1B
lovastatin
TCGA_PRAD
ICGC_PRAD_CA_seq
GSE70769
GSE107299
ICGC_PRAD_CA_array
GSE70768
DKFZ2018
GSE54460
GSE21034
GSE111177
broxaterol
albuterol
GSK1904529A
pimobendan
thiamphenicol
eprinomectin
3'−fluorobenzylspiperone
sulfameter
benztropine−mesylate
tecastemizole
vonoprazan
GW−9662
SKI−II
megestrol−acetate.1
linsitinib
dronedarone
manidipine
edrophonium
amitriptyline
AM−630
azeliragon
taprenepag
SGI−1027
propantheline
pinaverium
palovarotene
foropafant
diaveridine
L−165041
AZD1208
emedastine
azasetron
alendronate
norethindrone−acetate
5−fluoro−3−pyridyl−methanol
novobiocin.1
PD−128907.1
dimercaprol
letosteine
MK−3697
CFTRinh−172
GSK0660
EBPC
H−89
compound−401
phenelzine
ondansetron
AM−1241
captamine
creatinol−phosphate
RS−100329
birinapant
AM095
gemcadiol
piperacetazine
IEM1754
cefpirome
mesulergine
loxistatin−acid.1
CI−844
GSE70769
TCGA_PRAD
GSE111177
ICGC_PRAD_CA_array
GSE107299
ICGC_PRAD_CA_seq
GSE21034
GSE54460
GSE70768
DKFZ2018
Correlation
1
0.5
0
−0.5
−1

Fig 6. A. Mutation landscape of PCa between TAPBP^{high} and TAPBP^{low} subtypes. * $p < 0.05$, ** $p < 0.01$, *** $p < 0.001$, **** $p < 0.0001$. **B.** Drug sensitivity difference among different databases. Correlation of IC50 of molecular drugs and TAPBP expression levels across different databases in the GDSC database, CTRP database, and PRISM database.

3.9 Potential prostate cancer-causing proteins with clinical associations

In furtherance of the understanding of the clinical association of TAPBP, a number of specific PCa clinical indicators were used to correlate studies with it. As the most commonly used PCa screening indicator, PSA was first used for analysis. We found that the expression levels of TAPBP were not significantly associated with normal PSA (**Figure 7A**). Biopsy is the gold standard for PCa diagnosis, and Gleason score is usually used to determine the benignity or malignancy of biopsied tissue. Our study found that as the Gleason score increased, the expression level of TAPBP decreased (**Figure 7B**). Correspondingly TAPBP were more expressed overall in normal tissues than in tumor tissues (**Figure 7C**). Further analysis of the relationship between PCa TNM staging and the expression of TAPBP showed a negative correlation between the relative expression of TAPBP and TNM staging (**Figure 7D, E, F**), and showed lower expression in metastatic tumors (**Figure 7G**). Tumor mutational burden (TMB), as an emerging biomarker, has received increasing attention for its role in predicting the efficacy of tumor immunotherapy. High GWAS scores are associated with low TMB (**Figure 7H**).

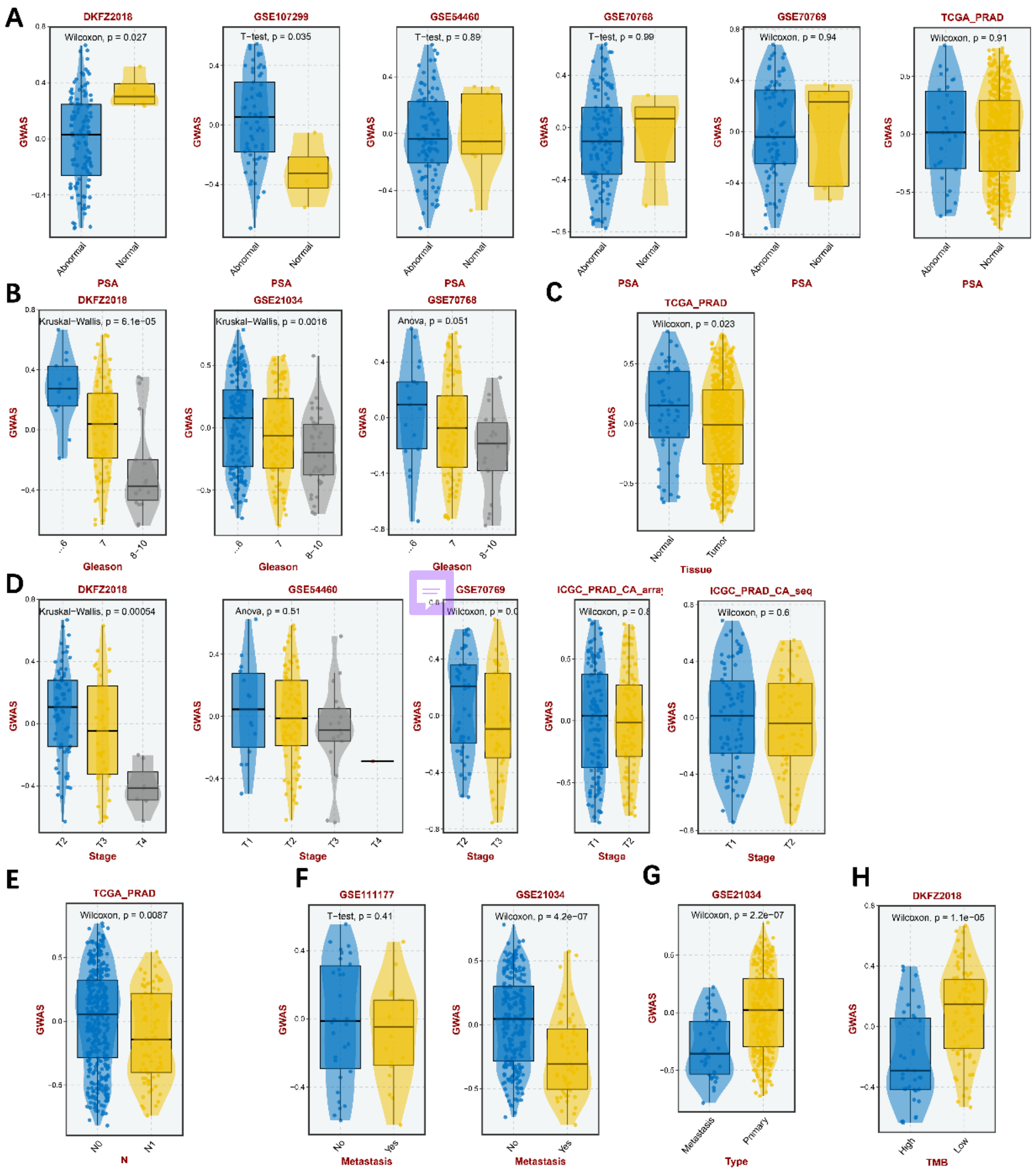

Figure 7. The role of TAPBP in PCa patient clinical outcomes. **A**. Relationship between TAPBP expression and PSA level in different databases. **B**. Relationship between TAPBP expression and Gleason score in different databases. **C**. Relationship between TAPBP expression and tissue type in TCGA database. **D, E, F.** Relationship between TAPBP expression and tumor TNM staging in different databases. **G.** Relationship between TAPBP expression in relation to tumor primary foci and metastatic foci. **H.** Relationship between TAPBP expression and TMB in DKFZ2018 database.

Discussion

Identifying therapeutic targets for disease is an important goal of human genetics research and is particularly important for PCA. In this study, we used a series of analytical techniques to investigate the functional associations between protein biomarkers in the prostate and PCa. We initially identified eight potential risk genes for PCa after PWAS analysis and FDR correction. Subsequently, we performed single-cell analysis of these genes and showed that they were differentially expressed in prostate. The candidate genes were validated in all of the above analyses and further enrichment analyses showed that it is primarily involved in immune response and cell cycle regulation. Further transcriptome association analysis revealed that four of the eight genes (CHMP2B, ATF6B, TAPBP, GAS1) were associated with PCa. Our results were replicated in MR validation analyses and verified whether the genes were risk factors or protective factors in the pathogenesis of PCa, providing a higher level of confidence. In addition, we identified MSMB, TAPBP and EGF by co-localization. Interestingly TAPBP was validated in all of the above analyses. Mendelian randomization analysis and clinically relevant analysis both suggest that TAPBP may be a protective factor for PCa. Therefore, we will further explore the mechanism of TAPBP in PCa in subsequent experiments.

TAPBP gene is close to the MHC and encodes a molecule that is a member of the IgSF. Its product, Tapasin, is required for the association of the MHC class I heterodimer with the Tap transporter protein in the endoplasmic reticulum (ER) [31]. It forms part of the peptide loading complex, which is essential for the stable assembly of class I molecules with peptides prior to transport to the cell surface.The TAPBP gene is located in the extended filamentous MHC region between the BING1 and RGL2 loci [32] .

Our study has several strengths. First, PWAS for PCa was performed using the largest and most comprehensive pooled statistics from the human proteome and the most recent GWAS for PCa. Second, we performed single-cell analyses as well as enrichment analyses of the genes identified by PWAS to further validate their expression in tissues and the molecular functions that the gene products may exercise, the cellular environments in which they reside, and the biological processes in which they are involved. Third, we validated the risk proteins using independent MR validation analyses. Fourth, based on Bayesian co-localization used to estimate the probability of observing

two correlated signals at specific loci with common causal variants, we confirmed the causative proteins of PCA (MSMB, TAPBP, and EGF). Finally, we explored the drug sensitivity and some clinical correlations of TAPBP, and the results indicated that low TAPBP is associated with worse clinical outcomes.

There are also some limitations to this study. First, the ARIC used in this study failed to incorporate the PSA protein. Second, including methylation data in the analysis could provide a more comprehensive picture of disease progression. Most of our studies were conducted in Europeans, but we should be careful to generalize these findings to other races. Last, the complexity of PCA biology and the molecular mechanisms behind it can only be understood through functional genomics approaches and biological experiments. Therefore, we will conduct more molecular biology experiments to validate our database-based findings.

conclusion

In conclusion, we have identified a number of candidate genes associated with PCa through a series of methods including PWAS, TWAS, MR, and Bayesian colocalization. They may be involved in the occurrence of PCa through immune regulation and cell cycle participation. The integrated results provide strong evidence for the association between TAPBP and PCa, and suggest that TAPBP may be a protective factor for PCa.

Author Contributions

XLW and AMJ: analyzing, writing-original draft, review, and editing. JMS: grammar correction. LW and ZYL: topic guidance and manuscript review.

Competing interests

The authors declare that they have no competing interests.

Ethics approval and consent to participate

The ethics of this study observe the Helsinki Declaration. Ethics approval was obtained from the Ethics Committee of Shanghai Changhai Hospital (No. CHEC2021-086). Human Ethics and Consent to Participate declarations: not applicable

Data availability

All data generated or analyzed during this study are included in this published article and its supplementary information files. Consult the corresponding author (ZYL) for raw data.

Funding

This work was supported by the 234 Panfeng Program at Changhai Hospital (No. 2020YXK030).

Reference

1. Sung H, Ferlay J, Siegel RL, et al. Global Cancer Statistics 2020: GLOBOCAN Estimates of Incidence and Mortality Worldwide for 36 Cancers in 185 Countries. CA Cancer J Clin. 2021 May;71(3):209-249.
2. Schumacher FR, Al Olama AA, Berndt SI, et al. Association analyses of more than 140,000 men identify 63 new prostate cancer susceptibility loci. Nat Genet. 2018 Jul;50(7):928-936.
3. Loh PR, Tucker G, Bulik-Sullivan BK, et al. Efficient Bayesian mixed-model analysis increases association power in large cohorts. Nat Genet. 2015 Mar;47(3):284-90.
4. Chang CC, Chow CC, Tellier LC, et al. Second-generation PLINK: rising to the challenge of larger and richer datasets. Gigascience. 2015 Feb 25; 4: 7.
5. Buniello A, MacArthur JAL, Cerezo M, et al. The NHGRI-EBI GWAS Catalog of published genome-wide association studies, targeted arrays and summary statistics 2019. Nucleic Acids Res. 2019 Jan 8;47(D1): D1005-D1012.
6. Visscher PM, Wray NR, Zhang Q, et al. 10 Years of GWAS Discovery: Biology, Function, and Translation. Am J Hum Genet. 2017 Jul 6;101(1):5-22.

7. Korte A, Farlow A. The advantages and limitations of trait analysis with GWAS: a review. Plant Methods. 2013 Jul 22; 9: 29.
8. Manolio TA, Collins FS, Cox NJ, et al. Finding the missing heritability of complex diseases. Nature. 2009 Oct 8;461(7265):747-53.
9. Sharma K, Schmitt S, Bergner CG, T et al. Cell type- and brain region-resolved mouse brain proteome. Nat Neurosci. 2015 Dec;18(12):1819-31.
10. Vogel C, Marcotte EM. Insights into the regulation of protein abundance from proteomic and transcriptomic analyses. Nat Rev Genet. 2012 Mar 13;13(4):227-32.
11. Rolland DCM, Basrur V, Jeon YK, et al. Functional proteogenomics reveals biomarkers and therapeutic targets in lymphomas. Proc Natl Acad Sci U S A. 2017 Jun 20;114(25):6581-6586.
12. Intasqui P, Bertolla RP, Sadi MV. Prostate cancer proteomics: clinically useful protein biomarkers and future perspectives. Expert Rev Proteomics. 2018 Jan;15(1):65-79.
13. Zhang J, Dutta D, Köttgen A, et al. Plasma proteome analyses in individuals of European and African ancestry identify cis-pQTLs and models for proteome-wide association studies. Nat Genet. 2022 May;54(5):593-602.
14. GTEx Consortium. Human genomics. The Genotype-Tissue Expression (GTEx) pilot analysis: multitissue gene regulation in humans. Science. 2015 May 8;348(6235):648-60.
15. GTEx Consortium. Erratum: Genetic effects on gene expression across human tissues. Nature. 2018 Jan 25;553(7689):530.
16. Melé M, Ferreira PG, Reverter F, et al. The human transcriptome across tissues and individuals. Science. 2015 May 8;348(6235):660-5.
17. Nieuwenhuis TO, Rosenberg AZ, McCall MN, et al. Tissue, age, sex, and disease patterns of matrisome expression in GTEx transcriptome data. Sci Rep. 2021 Nov 3;11(1):21549.
18. Gusev A, Ko A, Shi H, et al. Integrative approaches for large-scale transcriptome-wide association studies. Nat Genet. 2016 Mar;48(3):245-52.
19. Jiang A, Zhou Y, Gong W, et al. CCNA2 as an Immunological Biomarker Encompassing Tumor Microenvironment and Therapeutic Response in Multiple Cancer Types. Oxid Med Cell Longev. 2022 Mar 31; 2022: 5910575.
20. Bao Y, Jiang A, Dong K, et al. DDX39 as a predictor of clinical prognosis and immune checkpoint therapy efficacy in patients with clear cell renal cell carcinoma. Int J Biol Sci. 2021

Jul 25;17(12):3158-3172.

21. Jiang A, Bao Y, Wang A, et al. Establishment of a Prognostic Prediction and Drug Selection Model for Patients with Clear Cell Renal Cell Carcinoma by Multiomics Data Analysis. Oxid Med Cell Longev. 2022 Jan 4; 2022: 3617775.
22. Fromer M, Roussos P, Sieberts SK, et al. Gene expression elucidates functional impact of polygenic risk for schizophrenia. Nat Neurosci. 2016 Nov;19(11):1442-1453.
23. Walker VM, Zheng J, Gaunt TR, Smith GD. Phenotypic Causal Inference Using Genome-Wide Association Study Data: Mendelian Randomization and Beyond. Annu Rev Biomed Data Sci. 2022 Aug 10; 5: 1-17.
24. Boehm FJ, Zhou X. Statistical methods for Mendelian randomization in genome-wide association studies: A review. Comput Struct Biotechnol J. 2022 May 14; 20: 2338-2351.
25. Giambartolomei C, Vukcevic D, Schadt EE, et al. Bayesian test for colocalisation between pairs of genetic association studies using summary statistics. PLoS Genet. 2014 May 15;10(5): e1004383.
26. Schmidt AW, Kühnapfel A, Kirsten H, et al. Colocalization analysis of pancreas eQTLs with risk loci from alcoholic and novel non-alcoholic chronic pancreatitis GWAS suggests potential disease causing mechanisms. Pancreatology. 2022 May;22(4):449-456.
27. Colaprico A, Silva TC, Olsen C, et al. TCGAbiolinks: an R/Bioconductor package for integrative analysis of TCGA data. Nucleic Acids Res. 2016 May 5;44(8): e71.
28. Hänzelmann S, Castelo R, Guinney J. GSVA: gene set variation analysis for microarray and RNA-seq data. BMC Bioinformatics. 2013 Jan 16; 14: 7.
29. Cokelaer T, Chen E, Iorio F, et al. GDSCTools for mining pharmacogenomic interactions in cancer. Bioinformatics. 2018 Apr 1;34(7):1226-1228.
30. Geeleher P, Cox N, Huang RS. pRRophetic: an R package for prediction of clinical chemotherapeutic response from tumor gene expression levels. PLoS One. 2014 Sep 17;9(9):e107468.
31. Herberg JA, Sgouros J, Jones T, et al. Genomic analysis of the Tapasin gene, located close to the TAP loci in the MHC. Eur J Immunol. 1998 Feb;28(2):459-67.
32. Teng MS, Stephens R, Du Pasquier L, et al. A human TAPBP (TAPASIN)-related gene, TAPBP-R. Eur J Immunol. 2002 Apr;32(4):1059-68.

Click here to access/download
Supporting Information
STROBE-MR-checklist.docx

Click here to access/download
Supporting Information - Compressed/ZIP File Archive
MR supplement.zip
