# Supplementary material for: Multi-omics analysis indicates an association between TAPBP and prostate cancer
Source: PLoS One. 2025 Nov 21;20(11):e0336438. doi: 10.1371/journal.pone.0336438 (PMC12637951; doi:10.1371/journal.pone.0336438)
Supplement: Attachment — Submitted filename: Responses to reviewers.docx [file pone.0336438.s005.docx]

**Responses to editors’comments:**

Thank you for the editor’s kind suggestions. According to the editor’s requirements, we have endeavored to make the manuscript conform to the style of PLOS ONE and have included a funding statement in the new cover letter.

**Responses to reviewers’comments:**

**Major points of Reviewer #1**

Relevant paper with high level of genomic-related in silico analyses. Authors showcased experience in the field of cancer genomics and modeling. However, I would recommend referring to the work of Rosalind Eeles, world expert in prostate cancer causing genes. I do not see even one of her many seminal papers cited here. So I urge the authors to amend this manuscript by referring to studies of RE, who identified prostate cancer susceptibility genes and led world-wide studies on thousands of both healthy men and individuals with prostate cancer.

**Response:** We thank the reviewer for this meaningful suggestion. In the introduction section of the manuscript, we mentioned the research of Rosalind Eeles (Reference 2). Following the reviewer's suggestion, we have added a discussion of the similarities and differences with the study of Rosalind Eeles in the discussion section and provided a citation (Conti DV, Darst BF, Moss LC, et al. Trans-ancestry genome-wide association meta-analysis of prostate cancer identifies new susceptibility loci and informs genetic risk prediction. Nature genetics. 2021 Jan;53(1):65-75).

**Major points of Reviewer #2**

The manuscript titled “Multi-omics association study identifies potential prostate cancer-causing genes” presents a multi-omics approach to identify genes potentially associated with prostate cancer, with the aim of uncovering novel biomarkers. The study is well-executed, and the results are clearly presented. However, the discussion section is notably weak and requires substantial revision. It barely engages with the study’s own findings, limiting the interpretation and contextualization of the data. Additionally, the manuscript’s title is misleading, as the results do not fully support the strong claim implied. While the study shows promise, significant improvements—particularly in the discussion—are necessary to strengthen the manuscript. Additional suggestions are provided in the annotated PDF.

**Response:** We thank the reviewer for this meaningful suggestion. In the revised manuscript, we have provided a more in-depth explanation and discussion of the genes ultimately identified. Additionally, we have changed the title of the manuscript to “Multi-omics analysis indicates an association between TAPBP and prostate cancer.”
